# Exploring the Therapeutic Potenital of the Leaderless Enterocins K1 and EJ97 in the Treatment of Vancomycin-Resistant Enterococcal Infection

**DOI:** 10.3389/fmicb.2021.649339

**Published:** 2021-02-17

**Authors:** Ingvild Reinseth, Hanne H. Tønnesen, Harald Carlsen, Dzung B. Diep

**Affiliations:** ^1^Faculty of Chemistry, Biotechnology and Food Science, Norwegian University of Life Sciences, Ås, Norway; ^2^Section of Pharmaceutics and Social Pharmacy, Department of Pharmacy, University of Oslo, Blindern, Oslo, Norway

**Keywords:** bacteriocin, VRE infection, Eep, enterococci, EntK1, EntEJ97

## Abstract

The membrane-bound protease Eep is an important virulence factor in pathogenic enterococci. The protein is involved in stress response via the RIP pathway which is crucial for pathogenic enterococci to evade host immune attacks during infection. Eep serves also as a receptor for the bacteriocins enterocin K1 and enterocin EJ97. The bacteriocins kill *Enterococcus faecium* and *E. faecalis*, respectively, and their antibiotic resistant derivatives including vancomycin resistant enterococci (VRE). This functional duality of Eep makes these two enterocins very promising as options in the prospective treatment of enterococcal infections because wildtype enterococcal cells (with an intact Eep) are sensitive to the bacteriocins while bacteriocin-resistant-mutants (without a functional Eep) become less virulent. As a first step to explore their therapeutic potential in the treatment of systemic enterococcal infections, we investigated the compatibility of the bacteriocins with human blood, and the phenotypic changes of *eep*-mutants toward different stress conditions. We found that the bacteriocins were compatible with blood, as they did not cause haemolysis and that the bacteriocins retained most of their antibacterial effect when incubated in blood. The bacteriocins were autoclavable which is a crucial criterium for the development of parenteral administration. *Eep*-mutants, which became resistant to the bacteriocin were, as expected, less capable to withstand stress conditions such as exposure to lysozyme and desiccation. Further, their ability to chain, a trait implicated in niche adaptation as well as being necessary for genetic transfer via conjugation, was also severely affected. Together, these results indicate that the bacteriocins are promising for treatment of VRE infection.

## Introduction

Enterococci have emerged from the commensal enterococci to become a significant opportunistic pathogen and are especially dangerous for immunocompromised individuals (Lam et al., 2012; Rathnayake et al., 2012; Comerlato et al., 2013; Hefazi et al., 2016). They pose a serious problem not only due to their high innate resistance toward many antibiotics but also their well-developed ability to acquire resistance genes via genetic transfer, including resistance to vancomycin, a last resort antibiotic. Vancomycin resistant enterococci (VRE) have become a global health threat and the WHO has characterised VRE as a priority pathogen (Buetti et al., 2019). Studies have shown that when residing in the gastrointestinal tract of a patient treated with antibiotic, VRE can colonise and dominate the microbiota before disseminating to the blood stream (Donskey, 2004; Lam et al., 2012; Rosko et al., 2014). There they may cause sepsis and secondary infections including endocarditis (Donskey, 2004; O’Driscoll and Crank, 2015). Immunocompromised patients, such as cancer patients, are often given prophylactic antibiotic treatments, thereby creating an optimal condition for the opportunistic enterococci. Enterococci are also adapted to survival on biotic and abiotic surfaces and can disseminate from there after months of desiccation (Patel and Gallagher, 2015). This is especially problematic in hospital environments, where they can easily spread from surfaces or on equipment. In addition, research has shown that the immensely adaptable enterococci can become more resistant to alcohol after repeated exposure, possibly making modern sterilising methods insufficient in the future (Pidot et al., 2018). This increasing resilience behaviour coupled to the fact that there are no new antibiotic in development because of low profitability as a consequence of rapid resistance development, warrants for an acute need for new treatments to counteract VRE infections (Ovchinnikov et al., 2017).

In recent years, much attention has been attracted to a group of antimicrobial peptides, so-called bacteriocins, because they display potent activity toward important pathogens, including enterococci and their antibiotic-resistant derivates (Ovchinnikov et al., 2017; Reinseth et al., 2020). Bacteriocins are ribosomally synthesised peptides, produced by both Gram-positive and Gram-negative bacteria. Many bacteriocins of Gram-positive origin are relatively small peptides of size between 30 and 60 amino acids, and they often act as membrane-active and receptor mediated peptides that form pores on target membrane and cause death because of disrupted membrane integrity (Cotter et al., 2013; Simons et al., 2020).

The enterococcal bacteriocins enterocin K1 (EntK1) and enterocin EJ97 (EntEJ97) are promising antimicrobials against pathogenic enterococci as they pose potent activity toward *E. faecium* and *E. faecalis*, respectively. EntK1 is produced by *E. faecium* while EntEJ97 by *E. faecalis*, and they are most active within their producing species. The two bacteriocins are of the same length (37 amino acids) and share significant sequence similarity (over 50% identities) including a conserved motif (PWE) in their C terminal domains (PWE) which is important for the antimicrobial activity (Ovchinnikov et al., 2014). Both bacteriocins belong to the LsbB family (Ovchinnikov et al., 2017), which are known to specifically bind to Eep, a membrane-bound zinc metalloprotease on target cells (Uzelac et al., 2013). Even though Eep is conserved in *E. faecalis* and *E. faecium* there are subtle sequence differences that somehow confer different sensitivity of these two species to the bacteriocins. Their mode of action has not been fully determined yet, but it has been suggested that these bacteriocins create a pore in the membrane and subsequently cause cell death (Ovchinnikov et al., 2017). Evasion of the bacteriocins is therefore only possible through depletion of the receptor (by mutations).

Eep has been reported to be a virulence factor in several studies, through being involved in a stress response pathway that protects the bacteria from stress conditions encountered at an infection site, such as pH changes, reactive oxygen species and exposure to lysozyme (Le Jeune et al., 2010; Frank et al., 2013, 2015; Varahan et al., 2013). Eep, which is also called RseP, is part of a regulatory cascade, normally referred to as the regulated intramembrane proteolysis (RIP) pathway, that regulates the release of an extracytoplasmic sigma protein called SigV. SigV regulates stress responses in *E. faecalis* (Ovchinnikov et al., 2017). Therefore, resistance to the enterocins by lacking a functional Eep makes the bacteria less virulent, because they no longer have a functional stress-response and virulence factor—Eep.

To explore their therapeutic potential in VRE treatments, we present here a pre-clinical assessment of EntK1 and EntEJ97. The study includes the assessment of their compatibility with blood biomatrices, as well as an *in vitro* phenotypic characterisation of bacteriocin-induced *eep*-mutants. These mutants differ from previous works on Eep, because they are not bioengineered, but selected after exposure to the bacteriocin, hence mimicking an *in vivo* treatment situation.

## Materials and Methods

### Bacterial Strains, and Culture Conditions

The bacterial strains are listed in Table 1. Unless otherwise specified the bacterial strains were grown in brain heart infusion (BHI) medium (Oxoid) at 37°C under aerobic conditions without shaking.

**TABLE 1:** Bacterial strains.

Strain	Origin	Source
***E. faecium***		

P21	Wildtype-Laboratory	Herranz et al., 2001
LMGT 20705	Wildtype-Laboratory	Ghent bacterial collection
42 ACA-D6 0237	Wildtype-Laboratory	LMG collection (Ås. Norway)
E1469	Wildtype-Nosocomial	Gift from Rob Willems
E3160	Wildtype-Nosocomial	Gift from Rob Willems
E3449	Wildtype-Nosocomial	Gift from Rob Willems
E6072	Wildtype-Nosocomial	Gift from Rob Willems
E6094	Wildtype-Nosocomial	Gift from Rob Willems
E6856	Wildtype-Nosocomial	Gift from Rob Willems
E7195	Wildtype-Nosocomial	Gift from Rob Willems
E7422	Wildtype-Nosocomial	Gift from Rob Willems
E7660	Wildtype-Nosocomial	Gift from Rob Willems
E8150	Wildtype-Nosocomial	Gift from Rob Willems
P21c.ins7	Mutant derived from P21	This work
P21c.ins23	Mutant derived from P21	This work
P21c.ins43	Mutant derived from P21	This work
P21c.G716A	Mutant derived from P21	This work
3160c.535del	Mutant derived from E3160	This work
6856c.ins104	Mutant derived from E6856	This work
7195c.ins68	Mutant derived from E7195	This work
7422c.G62A	Mutant derived from E7422	This work
7422c.C64A	Mutant derived from E7422	This work
7422c.522del	Mutant derived from E7422	This work

***E. faecalis***		

D6	Wildtype-Laboratory	Palmer et al., 2010
OG1RF	Wildtype-Laboratory	Bourgogne et al., 2008
62	Wildtype-Laboratory	Brede et al., 2011
Symbioflor	Wildtype-Commercial probiotic	Domann et al., 2007
MMH594	Wildtype-Nosocomial	Huycke et al., 1991
V583	Wildtype-Nosocomial	Sahm et al., 1989
Merz96	Wildtype-Nosocomial	Harrington et al., 2004
T3	Wildtype-Nosocomial	Maekawa et al., 1992
TX0104	Wildtype-Nosocomial	The Nih Hmp Working Group et al., 2009
HH22	Wildtype-Nosocomial	Murray and Mederski-Samaroj, 1983
D6c.dup708	Mutant derived from D6	This work
T3c.del708	Mutant derived from T3	This work
TX0104c.C625A	Mutant derived from TX0104	This work

### Bacteriocins and Antimicrobial Assay

Synthetic enterocins K1 and EJ97 peptides were purchased from Pepmic Co., Ltd., China with 99% purity and solubilised to concentrations 1–10 mg/ml in Milli-Q water with 0.1% TFA. The peptides were stored at −20°C until use. The amino acid sequences of the bacteriocins are MLAKIKAMIKKFPNPYTLAAKLTTYEINWYKQQYGRYPWE RPVA and MKFKFNPTGTIVKKLTQYEIAWFKNKHGYYPWE IPRC for EntEJ97 and EntK1, respectively (Gálvez et al., 1998; Ovchinnikov et al., 2017). Bacteriocin activity was determined using a microtiter assay as previously described (Holo et al., 1991). MIC was determined as the minimum concentration that inhibited growth by at least 50% compared to control (without added bacteriocin) in 200 μl culture and the experiment was performed in three parallels.

### Haemolysis Assay

To assess the haemolytic effect whole blood was drawn from two healthy human volunteers, pooled and distributed in 1 ml aliquots. The aliquots were spun down at 1,000 × *g* for 5 min at 4°C to separate erythrocytes from plasma. The erythrocytes were washed three times with saline (0.9% NaCl) and resuspended in 100 μl saline. The isolated erythrocytes were then added to samples of 900 μl of the testing agent, as well as water for complete haemolysis and saline for the baseline, resulting in a 1 ml sample. SDS in saline was added as a control, since bacteriocins were solved in saline. The samples were incubated at 37°C for 1 h and then spun down again to remove intact erythrocytes. The supernatant was measured for absorbance at 540 nm in order to measure degree of haemolysis. Haemolysis is described as the percentage of lysed blood cells as calculated by $Haemolysis=\frac{Absorbance-Absorbance_{0}}{Absorbance_{100}}\times 100$, where Absorbance_0_ is the saline baseline and the Absorbance_100_ is complete haemolysis by water. The experiment was repeated three times to allow for statistical analysis.

### Antibacterial Effect in Blood

To test whether the antibacterial effect of the bacteriocins was reduced in blood, an assay was performed where whole blood was collected from two healthy volunteers, with EDTA as an anticoagulant. Directly after blood sampling each bacteriocin was added to a concentration of 1mg/ml. In parallel controls of ampicillin and saline in blood as well as bacteriocin in saline were prepared. The samples were placed at 37°C, except for one of the bacteriocin in saline samples which was left at room temperature to ensure that the incubation at 37°C itself did not diminish bacteriocin activity. Samples were collected at specific time points and bacteriocin activity was determined using the standard MIC assay as previously described (Holo et al., 1991). The experiment was repeated three times and the error bars represent the standard deviation.

### Generation and Verification of *E. faecalis* and *E. faecium Eep*-Mutants

Bacteriocin resistant mutants were obtained by a spot-on-lawn assay, where liquid bacteriocin was spotted on a lawn of indicator cells, using 10μl of bacteriocin in concentrations 0.5–5 mg/ml. The plates were incubated at 30°C overnight and resistant colonies were selected from the inhibition zones and re-streaked on new plates for isolated colonies. Pure cultures were made and stored in 15% glycerol at −80°C. The *eep* gene was PCR amplified and sequenced with specific primers: *eep* in *E. faecium* was amplified using primers Eepf4F + Eepf4R and sequenced using primers Eepf1R - Eepf6R and Eepf1F - Eepf6F. *eep* in *E. faecalis* was amplified using primers Eepfa1R + Eepfa3F and sequenced using primers Eepfa1R - Eepfa3R, Eepf1R - Eepf3R and Eepfa1F - Eepfa3F. All primers are listed in Table 2.

**TABLE 2:** Oligonucleotide primers.

Primer	Oligonucleotide sequence (5′ to 3′)
Eepf1R	GACAGTTTGCTGTTTGTATTCCC
Eepf2R	TGTTCATGAGTTTGGTCATTTC
Eepf3R	CTCTTTTACTAAATTCTGACG
Eepf4R	AGTTAAGTCCTGATATTTCG
Eepf5R	TTAAGTCCTGATATTTCGC
Eepf6R	TCTTTGAAACGATACAAGTT
Eepf1F	CGAAGCGTATATGTTGTCCCGT
Eepf2F	TAAAACCTGTATTCATTGGG
Eepf3F	GCTCTTAGCAAGATTTGATGGC
Eepf4F	GATATGAATAAATACCCGCT
Eepf5F	GCTAATGGCAGATATGAAT
Eepf6F	GAATTTCATAAAAGACGCAG
Eepfa1R	TAGGCGAAGTGGTCAAGTCC
Eepfa2R	TTTTACGAGACTTTCCCATGT
Eepfa3R	ATTCTGTTTACGTTAGCGG
Eepfa1F	TTTCATATAAGGATAAACGCCGACT
Eepfa2F	CTTCTGCATCATTTGGTACTTC
Eepfa3F	GGTTTCTTCATGCGTTGGGC

The samples were sent to GATC Biotech, Germany for sequencing. MIC of the resistant mutants was scored using a microtiter assay as previously described (Holo et al., 1991).

### Tolerance Assays

To test for lysozyme tolerance the wild type and mutant strains were grown over night and subjected to a standard MIC assay as previously described with twofold dilutions of lysozyme (Holo et al., 1991). In addition, a spot-on-lawn assay was performed with 10 μl 250 mg/ml lysozyme spotted on a lawn of cells.

To test for differences in desiccation survival between the *eep*-mutants and wildtype, a desiccation survival assay was performed as follows. Overnight culture was distributed in the first column of a 96-well microtiter plate, with 20 μl in each well. These plates were left to dry at room temperature in natural light. At specific time points samples were added fresh media and serial dilutions were made. From these dilutions, samples were plated, incubated overnight and CFU was enumerated as a measure of desiccation survival.

### Scanning Electron Microscopy (SEM)

Strains were inoculated in fresh BHI medium and grown at 37°C until OD_550_ = 0.3. Strains were grown in duplicates where one was added 500 μl of 200 mg/ml lysozyme to a final concentration of 20 mg/ml, and the other was added 500 μl BHI. The strains were then mixed 1:1 with the fixation solution, 4% paraformaldehyde (w/v) + 5% glutaraldehyd (v/v) in 1× PBS and left at 4°C over night. The samples were then washed with 0.2 M CaCo buffer and dehydrated before analysis by SEM.

## Results

### Antibacterial Effect of EntK1 and EntEJ97

EntK1 and EntEJ97 specifically target Eep in enterococci (Ovchinnikov et al., 2017). We wanted to compare the potency of these bacteriocins toward laboratory and nosocomial strains. As shown in Table 3, most of these strains were relatively sensitive to the bacteriocins, regardless whether they were of laboratory type or nosocomial type. Toward the *E. faecium* strains MIC varied from <0.048 to 0.78 μg/ml, with strains P21 and 42 ACA-D6 0237 being the most sensitive (both with MIC less than 0.048 μg/ml) while E8159 being the least sensitive (with MIC of about 0.78 μg/ml). Toward the *E. faecalis* strains, four of the six strains had MIC between 0.39 and 0.78 μg/ml, one had MIC just above 1 μg/ml, while the last one, strain V583, appeared to be resistant as a concentration of 25 μg/ml did not kill the strain. When examining the genome of V583 we identified a gene encoding the immunity protein of enterocin EntEJ97 (data not shown); the presence of this gene likely explains the resistance of V583 toward the bacteriocin.

**TABLE 3:** The minimal inhibitory concentration (MIC) of EntK1 and EntEJ97 against laboratory (L), nosocomial (N), and probiotic (B) strains of *E. faecium* and *E. faecalis*.

*E. faecium* strain	MIC_μ__g/ml_ of EntK1
P21 (L)	<0.048
LMGT 20705 (L)	<0.048
42ACA-D6 0237 (L)	0.19
E1469 (N)	0.39
E3160 (N)	0.39
E3449 (N)	0.39
E6072 (N)	0.19
E6094 (N)	0.39
E6856 (N)	0.39
E7195 (N)	0.39
E7422 (N)	0.19
E7660 (N)	0.39
E8150 (N)	0.78

***E. faecalis* strain**	**MIC_μ__g/ml_ of EntEJ97**

D6 (L)	0.39
OG1RF (L)	0.78
62 (L)	1.56
Symbioflor (B)	0.39
MMH594 (N)	0.39
V583 (N)	>25
Merz96 (N)	0.78
T3 (N)	0.39
TX0104 (N)	0.39
HH22 (N)	1.56

### Haemolytic Activity of the Bacteriocins in Blood

**FIGURE 1:**
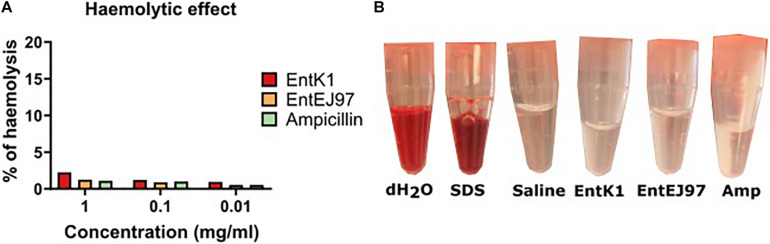
Compatibility of the bacteriocins with blood. Whole human blood was mixed with each bacteriocin in saline to a final concentration of 1, 0.1, and 0.01 mg/ml. The samples were incubated at 37°C before the erythrocytes were spun down and the supernatant was read at OD_540_. **(A)** Percentage of haemolysis after contact with EntK1, EntEJ97 and Ampicillin at 1, 0.1, and 0.01 mg/ml. **(B)** Visual haemolysis after contact with dH_2_O, 0.1% SDS in saline. saline, and 1 mg/ml EntK1, 1 mg/ml EntEJ97, and 1 mg/ml Ampicillin. Like dH_2_O 0.1% SDS showed complete haemolysis, while 1 mg/ml EntK1, 1 mg/ml EntEJ97, and 1 mg/ml Ampicillin, all in saline, did not cause haemolysis as judged from the lack of redness, an indication of haemolysis, in the supernatant, compared to saline.

VRE cause a range of infections, such as endocarditis, meningitis, sepsis and urinary tract infections (O’Driscoll and Crank, 2015; Reinseth et al., 2020). To reach these infection sites the bacteriocin would need to be circulated systemically and interact with blood constituents without causing lysis of erythrocytes. The haemolytic effect of the bacteriocins was therefore evaluated. No obvious haemolysis in erythrocytes were observed at the concentrations tested (0.01, 0.1, and 1 mg/ml), which are much higher than the MICs for all tested strains (Figure 1). This result was also comparable to what was observed for ampicillin, which is an antibiotic used in intravenous antibacterial treatment.

As saline (0.9% NaCl) was used to dissolve the bacteriocins, we wanted to rule out the possibility that saline could have had an isotonic effect thereby preventing haemolysis by the bacteriocins. To test this, we challenged the blood cells with 0.1% SDS dissolved in saline. As seen in Figure 1 haemolysis was evident in the SDS solution, but not in the saline solution alone, confirming that the isotonic effect of saline could not prevent the haemolysis caused by SDS. In addition, we also confirmed that pure water, which is hypotonic, easily caused haemolysis of the blood cells.

### Antibacterial Activity of the Bacteriocins in Blood

**FIGURE 2:**
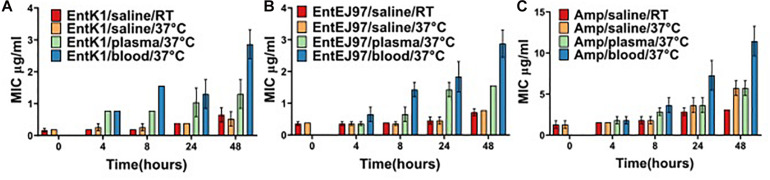
The MIC value (μg/ml) for EntK1 **(A)**, EntEJ97 **(B)**, and Ampicillin **(C)** during blood incubation. The presented MIC values are the average of three experiments. Each bacteriocin was added to whole human blood to reach a concentration of 1 mg/ml. The samples were incubated at 37°C and then checked for antibacterial activity at time points 4, 8, 24, and 48 h using a standard MIC assay. The results indicate a 2–4-fold reduction in bacteriocin activity over the course of the experiment. The same result presented for Ampicillin, which lost fourfold activity by 48 h. RT is short for room temperature.

The bacteriocins investigated in the current work are hydrophobic, possibly implicating interaction with several blood constituents (Gálvez et al., 1998; Ovchinnikov et al., 2017). If this interaction sequesters or somehow disturbs the antimicrobial effect of the bacteriocins, the suitability of the bacteriocins as an intravenous treatment would be compromised. Therefore, the antimicrobial effect of the bacteriocins was tested after incubation in whole blood and plasma (Figure 2). In saline the MIC of EntK1 was 0.19 μg/ml, and when added to blood or plasma the MIC was increased fourfold, to 0.78 μg/ml. After 8 h of incubation at 37°C the MIC of EntK1 in saline stayed at the same level, as did the MIC of EntK1 in plasma, which was 0.78 μg/ml. The MIC of EntK1 in blood, however, increased to approximately 1.56 μg/ml. After 24 and 48 h of incubation, the MIC increased in all groups, but at the same proportional rate. The MIC of EntK1 at 48 h in saline was 0.39 μg/ml, EntK1 in plasma 1.56 μg/ml and EntK1 in blood 3.125 μg/ml, but the ratios between them were the same at all time points (2× higher in plasma than saline, and 2× higher in blood than plasma).

The same ratio pattern was also observed for EntEJ97, with MIC 0.39 μg/ml in saline, 0.78 μg/ml in plasma and 1.56 μg/ml in blood after 8 h. After 48 h the MIC increased to 0.78 μg/ml in saline, 1.56 μg/ml in plasma and 3.125 μg/ml in blood.

Ampicillin was used as a control as this antibiotic is in use as an intravenous treatment against bacterial infections (Hellinger et al., 1992). Interestingly it seemed to follow the same pattern as the bacteriocins in terms of losing effect over time, starting at MIC 1.56 μg/ml in blood and ending with MIC 12.5 μg/ml at 48 h incubation.

Samples of the antimicrobials in saline left at room temperature (22–25°C) was included to ensure that the variation seen was due to the effect of the biomatrices alone and not the temperature at 37°C. At all time points samples at room temperature mirrored, though with some minor variations, that of the corresponding samples at 37°C, confirming the stability of the bacteriocin at 37°C.

To summarise, the MIC of the bacteriocins in blood and plasma increased by 2× or 4× from 4 to 48 h indicating only a 2–4-fold decrease in bacteriocin activity over 44 h. In addition, the MIC of the bacteriocins in blood compared to saline was 4× higher for EntK1 and 2× higher for EntEJ97. In comparison ampicillin appeared to follow the same pattern, loosing 4×–8× activity after 48 h incubation in similar conditions.

### Bacteriocin Activity Is Resilient to Autoclaving

**FIGURE 3:**
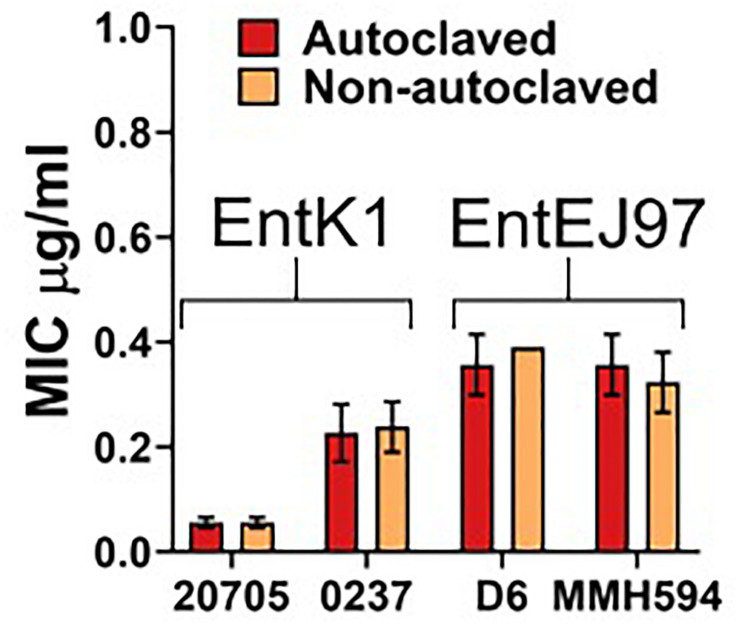
Effect of autoclaving on EntK1 and EntEJ97. Each bacteriocin was solved in MilliQ water with 0.1% TFA and then autoclaved. The antibacterial effect of EntK1 and EntEJ97 was then tested against two strains of *E. faecium* and two strains of *E. faecalis*, respectively, in a standard MIC assay. The resulting MIC values are comparable between the autoclaved and non-autoclaved samples, indicating that autoclaving did not affect the antimicrobial effect of the enterocins. Strains: 20705 is short for *E. faecium* LMGT 20705; 0237 for *E. faecium* 42 ACA-D6 0237; D6 for *E. faecalis* D6; and MMH594 for *E. faecalis* MMH594.

A pre-requisite for intravenous administration is complete sterility of the compound (Paese et al., 2017). The simplest manner to achieve this is through autoclaving. However, this can cause degradation of the compound and therefore be detrimental to the antibacterial effect. Also, if the compound is degraded into unknown constituents this may cause unintended interactions. Therefore, we exposed the bacteriocins to a standard autoclaving procedure. The results indicated that autoclaving had no detrimental effect on the bacteriocins as their antimicrobial activity was virtually the same before and after autoclaving (see Figure 3).

### Bacteriocin-Resistant Mutants Have Mutations Within *Eep*

**FIGURE 4:**
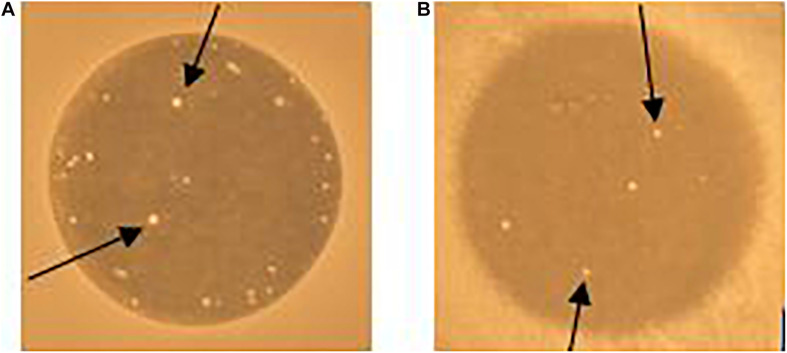
Resistant colonies (white spots) occur in EntK1 and EntEJ97 inhibition zones. 10 μl of 1 mg/ml EntK1 **(A)** or EntEJ97 **(B)** was applied onto soft agar containing *E. faecium* P21 **(A)** or *E. faecalis* MMH594 **(B)**. The plate was left overnight at 30°C for cell growth and inhibition of cells. Arrows indicate resistant colonies growing within the inhibition zones.

When enterococcal cells were exposed to EntK1 or EntEJ97 in the spot-on-lawn assay, we frequently observed resistant colonies in the inhibition zones (see Figure 4). Cells derived from these colonies had high MIC values for the bacteriocin used in the test, mostly above 25 μg/ml for both EntK1 and EntEJ97, in a cross-resistant manner. As the membrane bound protein Eep is known to serve as the receptor for EntK1, EntEJ97, and other bacteriocins belonging to the same family (Ovchinnikov et al., 2017), we suspected that these resistant cells might have acquired deleterious mutations within the *eep* gene. Random resistant cells were therefore collected and the *eep* gene sequenced. As expected, all the mutants contained mutations within *eep* which caused various outcomes, ranging from insertions to duplications and substitutions (see Table 4).

**TABLE 4:** Strain specific mutations.

Parent strain	Mutant strain and type of mutation	Gene position	Outcome
***E. faecium***			

P21	P21c.ins7—transposon insertion	7	Gene interruption
P21	P21c.ins23—transposon insertion	23	Gene interruption
P21	P21c.ins43—transposon insertion	43	Gene interruption
P21	P21c.G716A—base substitution	716	Early stop codon
E3160	3160c.535del—base deletion	535	Early stop codon
E6856	6856c.ins104—transposon insertion	104	Gene interruption
E7195	7195c.ins68—transposon insertion	68	Gene interruption
E7422	7422c.G62A—base substitution	62	Amino acid substitution
E7422	7422c.C64A—base substitution	64	Amino acid substitution
E7422	7422c.522del—base deletion	522	Early stop codon

***E. faecalis***			

D6	D6c.dup708—duplication	708	Frame shift
T3	T3c.del708—duplication	708	Frame shift
TX0104	TX0104c.C625A—base substitution	625	Early stop codon

#### Phenotypic Changes in Resistant Mutants

Considering that *E. faecium* is increasingly becoming more prevalent in medical conditions, and has higher levels of antibiotic resistance than *E. faecalis* and that most previous research on *eep*-mutants has only concerned *E. faecalis*, we therefore considered it appropriate to focus further characterisation on *E. faecium* (Frank et al., 2013, 2015; Varahan et al., 2013; Prematunge et al., 2016; Reinseth et al., 2020). As Eep is involved in stress response, the *eep*-mutants were analysed as to how they would cope with different stress factors. Lysozyme is one of the first defences of the body against pathogens, and therefore a deficiency in lysozyme resistance would reduce a pathogen’s virulence capacity. Two wild type strains, one of laboratory origin (P21) and one of nosocomial origin (E7422), and their respective mutants, were tested in a lysozyme MIC assay. As seen in Table 5 both wild type strains tolerated more lysozyme than their mutants, i.e., the MIC values for the wildtype strains being 6–8-fold higher than for the mutant strains, 15.63 and 0.98–1.95 mg/ml, respectively.

**TABLE 5:** Lysozyme MIC in wildtype (WT) and mutant (M) strains.

*E. faecium*	Lysozyme MIC mg/ml
P21 (WT)	15.63
P21c.ins7 (M)	0.98
P21c.ins23 (M)	0.98
P21c.ins43 (M)	0.98
P21c.G716A (M)	0.98
E7422 (WT)	15.63
7422c.C64A (M)	1.95
7422c.522del (M)	1.95

**FIGURE 5:**
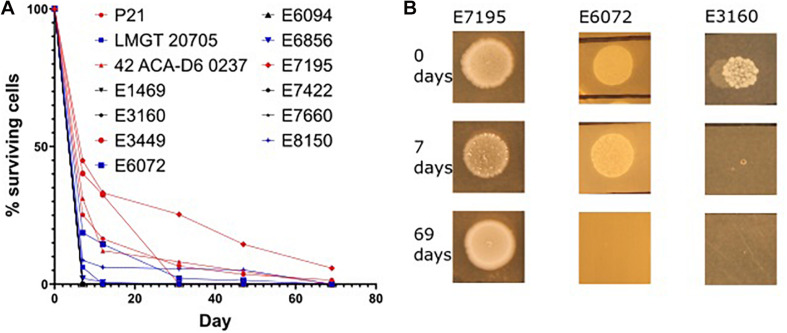
Desiccation variation in *E. faecium* strains. Overnight culture of each strain was placed in the first well of a microtiter plate and left for up to 69 days. At the specified time points fresh media was added, titrated and applied onto agar plates. After incubation at 30°C for 24 h the samples were enumerated by CFU counting. The percentage of live cells for the different strains at the different days is shown in **(A)**. Three desiccation phenotypes are presented in **(B)**. Some strains died before 7 days (represented by E3160), some died between day 7 and 69 (represented by E6072), and some persisted beyond day 69 (represented by E7195). There was no obvious correlation between origin, nosocomial or laboratory, and the amount of time the strain survived desiccation.

Enterococci can survive on surfaces for extended periods of time (Cetinkaya et al., 2000). This ability promotes the ability of the bacteria for spreading in e.g., hospital environments. Considering the role of Eep in stress response we hypothesised that it may also be implicated in desiccation survival. To our knowledge there is no data on desiccation survival on *E. faecium* (or *E. faecalis*) strains. Before testing the *eep*-mutants, we wanted to examine how different wild type strains responded to desiccation. Interestingly, as seen in Figure 5 three phenotypes presented among the wild type strains: the first indicated a rapid deterioration of cells and complete death 7 days from desiccation induction, as seen with *E. faecium* E3160. The second showed slow decrease in cell number and complete death by day 69, as seen with *E. faecium* E6072. The third exemplified by E7195 persisted beyond day 69. No obvious coherence between strain origin (nosocomial or laboratory) and desiccation tolerance was found.

**FIGURE 6:**
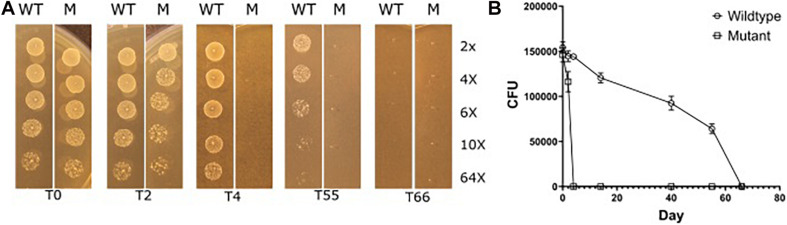
Reduced desiccation tolerance in the *eep* mutant P21c.ins7 (M) compared to the wildtype counterpart P21 (WT). Cells were exposed to desiccation and at the indicated days (day 0, 2, 4, 55, and 66) cells were diluted in a serial manner and spotted onto agar plates to estimate cell amounts. See “Materials and Methods” section for detail of the experiment. As seen in **(A)**, wild type cells survived between 55 and 66 days of desiccation while mutant cells disappeared after 4 days of desiccation. In **(B)**, cell survival is shown in CFUs which are means of three independent experiments.

We therefore subjected selected strains that had long desiccation survival phenotypes, *E. faecium* P21 and E7422, with their corresponding mutant strains (P21c.ins7 and 7422c.522del), to the same desiccation survival assay. The wildtype strains persisted at least to day 55. Strikingly, the *eep*-mutant strains were severely affected as judged from the fact that no live cells were found in the samples of these mutants from day 4 of desiccation. This result can be exemplified with the wild type *E. faecium* P21 and its *eep*-mutant P21c.ins7 in Figure 6.

### Scanning Electron Microscopy Reveals Loss of Chaining in *Eep*-Mutants

**FIGURE 7:**
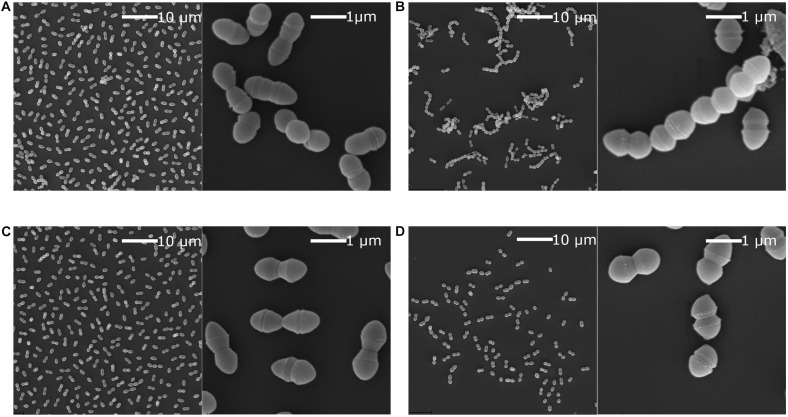
Scanning electron microscopy of *E. faecium* wildtype strain P21 and the *eep* mutant strain P21c.ins7. Scanning electron microscopy was performed in the presence or absence of 2 mg/ml lysozyme. Cultures were grown to OD_550_ at 0.3 and then mixed 1:1 with fixation solution. This was left overnight at 4°C before preparing the samples. Cells of the wildtype **(A)** and the mutant **(C)** cells were diplococcal when lysozyme was absent. When lysozyme was present, wild type cells were converted to longer chains of cells **(B)** while the mutants cells remained as diplococci **(D)**. Low cell number in **(D)** was likely due to lower tolerance to lysozyme in the mutant.

Chaining has been implicated in niche adaptation and genetic transfer by conjugation (Domenech et al., 2018; McKenney et al., 2019). Previously it has been shown that strains with a non-functional SigV, which is the Eep associated sigma factor, lose the ability to chain in *E. faecalis* (Varahan et al., 2013). We hypothesised that the same would be true for the *E. faecium eep*-mutants. SEM analysis was performed on P21 and E7422 wildtype and counterpart mutant strains (P21c.ins7 and 7422c.522del), with and without lysozyme stress. As expected, the wildtype strains had a diplococcal morphology when lysozyme was not present but formed chains when exposed to lysozyme. In contrast, the mutant strains were not able to chain when exposed to lysozyme. An example from this experiment is shown in Figure 7 for the wild type P21 and the mutant P21c.ins7.

## Discussion

Antimicrobial peptides have long been proposed as treatment options although traditionally they have not been sufficiently preclinically assessed. Among the arising and most serious multidrug resistant bacteria are the VRE. These bacteria are human commensals but are also optimally positioned for opportunistic infection, with significance in immunocompromised individuals. The bacteriocins EntK1 and EntEJ97 specifically target enterococci through the Eep protein in the bacterial membrane. Eep is a virulence factor and bacteria without Eep (i.e., *eep-*mutants) will therefore become less capable of responding to stress (e.g., immune responses in the human host) and consequently, also less capable of establishing infection. In this study we have shown that the enterocins are promising in preclinical evaluation. The bacteriocins had MIC values for different enterococcal strains ranging from 0.048 to 1.56 μg/ml, which is promising for reaching efficient treatment doses. However, several studies have reported caution when estimating therapeutic doses based on MIC values from *in vitro* settings (Levison and Levison, 2009; Wen et al., 2016). This should therefore be tested in a relevant *in vivo* model. The nosocomial strains did not consistently have higher MIC values than the laboratory strains, indicating that there was no obvious adaptation to EntK1/EntEJ97 resistance in strains isolated from infection sites. Both EntK1 and EntEJ97 were compatible with blood, as they did not cause haemolysis and only moderately decreased in antimicrobial effect, in an extent comparable to ampicillin, in human blood. The bacteriocins were autoclavable which is important to avoid bacterial contamination during drug formulation. *Eep*-mutants were less capable to withstand lysozyme and desiccation indicating that they had decreased fitness, hence likely also decreased virulence. The *eep*-mutants lost the ability to chain, further indicating loss of adaptation to stressful conditions.

An important goal of this study was to determine whether the bacteriocins were compatible with blood, which is required for the development of parenteral treatments (Paese et al., 2017). Most antimicrobial peptides are cationic and amphiphilic. Consequently, one of the main concerns with antimicrobial peptides are their ability to cause haemolysis, through positively charged amino acids binding to the negatively charged lipid bilayer. This binding can cause membrane disintegration and eventually erythrocyte-burst (Kumar et al., 2020). By analysis of the peptides with the haemolytic potential prediction programme HemoPred (Win et al., 2017), EntK1 is predicted to have haemolytic potential, while EntEJ97 is not. We have shown here that neither of the peptides are haemolytic up to a concentration of 1 mg/ml. The secondary structure of EntK1 consists of an α-helix from residue 8 to residue 25 (Ovchinnikov et al., 2017). Hydrophobic residues in an α-helix increases a peptide’s probability of being haemolytic (Win et al., 2017). In the region predicted to be α-helical EntK1 has 10 hydrophobic residues out of 18. Specific motifs and residues are important for haemolytic capability, and although EntK1 is predicted to be haemolytic it does not have any of the reported motifs. In addition, only 11 out of 37 residues are reported as important for haemolysis (L, K, F, and W) (Chaudhary et al., 2016; Oddo and Hansen, 2017). EntEJ97 has a total of 44 amino acids. Of them 22 are hydrophobic amino acids and 12 were predicted important for haemolysis. To our knowledge no structural studies have been performed on EntEJ97. Altogether this indicates that even though these bacteriocins are hydrophobic, they are non-haemolytic up to 1 mg/ml concentration in experimental settings. The fact that the peptides have some hydrophobic properties means they have potential to bind to serum proteins. It is possible that this binding could interfere with the antibacterial effect of the bacteriocins, but it is also possible that the binding could aid drug distribution. Svenson et al. (2007) and Sivertsen et al. (2013, 2014) found in two independent studies that a range of antibacterial peptides associated with the two major plasma proteins human serum albumin (HSA) and alpha-1 acid glycoprotein. However, only binding to HSA reduced the antibacterial effect of the peptides. In our study we investigated whether incubation in blood decreases the antibacterial effect of the bacteriocins. We found that there was some decrease in antibacterial effect over time, but that the MIC was consistently 2–4 times higher in blood compared to saline, including for ampicillin as control. This reduction in activity could be attributed to HSA binding, although this is unlikely because previous research has shown that the concentration of HSA is higher in plasma than in blood. It is also possible that the bacteriocin was degraded by blood constituents (Svenson et al., 2007; Bottger et al., 2017; Harm et al., 2019). Previous work has found that the stability of peptides is greater in whole blood than in plasma and serum (Bottger et al., 2017). This does not correspond to our study where the MIC values of our bacteriocins were consistently higher in blood than in plasma. This difference could be due to the nature of the peptides *per se*. However, it is also tempting to attribute this discrepancy to the bacteriocin associating to erythrocytes and therefore some of the antibacterial effect being quenched by this association. The MIC values showed promise for *in vivo* treatment. The bacteriocins were resilient to autoclaving as expected. Bacteriocins are mainly heat stable (Nes et al., 2014), and several studies have investigated AMPs from other lactic acid bacteria producers and found that they were resilient to autoclaving (Jamuna and Jeevaratnam, 2004; Chang et al., 2018).

Several studies have reported the involvement of SigV and Eep in enterococcal virulence. SigV, an extracytoplasmic sigma factor, is involved in enterococcal resistance toward different environmental stresses such as heat and acids (Benachour et al., 2005). SigV is also involved in lysozyme resistance, and in the ability of *E. faecalis* to cause urinary tract and intravenous infection in a murine model (Le Jeune et al., 2010). *eep* deletion-mutants were attenuated in an endocarditis rabbit model, and showed reduced kidney colonisation in a urinary tract infection model (Frank et al., 2012). The relationship between Eep and SigV was established by experiments that showed that an *eep* deletion mutant phenocopied a s*igV* deletion mutant in the resistance against heat, low pH and ethanol stress (Varahan et al., 2013). This, and similar studies of homologous systems in other species indicate that the Eep protein is involved in the breakdown of RsiV which is the anti-sigma factor to SigV (Benachour et al., 2005; Varahan et al., 2013; Ovchinnikov et al., 2017). Therefore, Eep likely controls the activation of the stress response that is transcribed from the *sigV* regulon. Several of these studies have proposed Eep and/or the SigV stress response as promising targets for antibacterial therapy (Le Jeune et al., 2010; Frank et al., 2013). Ovchinnikov et al. (2017) reported that in *E. faecalis* and *E. faecium* Eep is the receptor for EntK1 and EntEJ97, and that resistant mutants have a non-functional Eep protein. Most previous works on *eep*-mutants have been done with *E. faecalis* strains (Benachour et al., 2005; Le Jeune et al., 2010; Frank et al., 2012, 2013, 2015; Varahan et al., 2013). In this study we report *eep*-mutants of *E. faecium*. This opportunistic pathogen, although somewhat less virulent than *E. faecalis*, is more frequent in vancomycin resistance than *E. faecalis* and therefore becoming a major threat to public health (Comerlato et al., 2013; Kristich et al., 2014). *E. faecalis* is well-known for its lysozyme resistance, and although not well characterised there are some reports of lysozyme resistant *E. faecium* strains (Le Jeune et al., 2010; Kivanc et al., 2016). The *eep*-mutants of *E. faecium* in the present study showed an 8x reduction in lysozyme tolerance compared to the wild type strains. Lysozyme is a first line of defence in humans, important to reduce the load of invading microorganisms (Le Jeune et al., 2010). Our findings in *E. faecium* correlate well with previous findings that also report a reduction in lysozyme tolerance in *eep* or s*igV* mutants of *E. faecalis* (Benachour et al., 2005; Le Jeune et al., 2010).

Wild type enterococcal cells are extremely adapt to survival on surfaces and have been reported to persist for years. We report here that strains with mutations in *eep* have a dramatically decreased ability to survive desiccation. This is in line with a previous study which showed that the Eep-activated *sigV*-operon is overexpressed during exposure to starvation (Benachour et al., 2005). This finding is of utmost importance due to the spread of enterococci not only from biotic surfaces such as skin and hair but also from abiotic surfaces e.g., floors, ventilators and surgery equipment in hospitals. If the bacteriocin resistant *eep*-mutants are not able to survive on surfaces due to a failure in activating the appropriate stress response, treatments with the bacteriocins (e.g., as ingredients in wash or spray solution) could decrease enterococcal cells from surfaces and thereby limit this reservoir. However, this phenotype needs to be confirmed in more strains, and the possibility that other factors are causing the phenotype should be explored. Similarly *E. faecalis* switches from diplococci to long chain morphology, as a stress response upon exposure to bile salts (McKenney et al., 2019). Chaining has also been implicated in competence to exchange antibiotic resistance genes in other cocci (Domenech et al., 2018). This chaining adaptation has been reported to be faulty in *eep*-mutants of *E. faecalis* when exposed to lysozyme (Varahan et al., 2013). We found that the same was true for *E. faecium*, as its *eep*-mutants did not chain when exposed to lysozyme (see Figure 7). This is relevant for treatment considering that antimicrobial resistance can develop from repeated exposure in the gastrointestinal tract, and lysozyme is an important local innate defence (Dommett et al., 2005; Le Jeune et al., 2010). If the enterocin-treated enterococci (either being killed or become *eep*-mutants) are unable to adapt to the gastrointestinal environment, they can no longer persist and disseminate from there. As the gastrointestinal tract is an important reservoir for VRE infection in immunocompromised individuals, these results might be very useful when developing strategies to reduce risk of VRE in such medical conditions.

These results taken together could indicate that Eep is essential for enterococcal survival of several types of stressors commonly encountered in *in vivo* situations, and that bacteriocin-induced *eep*-mutants are likely less capable to establish infection. This, in addition to the lack of toxicity and compatibility with blood, makes the bacteriocins EntK1 and EntEJ97 ideal for treatment of VRE infections. However, this notion is valid only for the strains tested here, and future work should therefore investigate whether these traits are of general features for these two species and whether they are also applied to relevant *in vivo* models.

## Data Availability Statement

The original contributions presented in the study are included in the article/supplementary material, further inquiries can be directed to the corresponding author/s.

## Author Contributions

IR designed and performed the experiments, analysed the data, and wrote the manuscript. HT and HC designed, analysed the data, and revised the manuscript. DD obtained funding, designed the experiments, and revised the manuscript. All authors contributed to the article and approved the submitted version.

## Conflict of Interest

The authors declare that the research was conducted in the absence of any commercial or financial relationships that could be construed as a potential conflict of interest.
